# Evaluation of cardiac valvular regurgitant lesions by cardiac MRI sequences: comparison of a four valve semi-quantitative vs. quantitative approach

**DOI:** 10.1186/1532-429X-15-S1-P112

**Published:** 2013-01-30

**Authors:** Sahadev T Reddy, Mark Doyle, Moneal Shah, Diane A Vido, Ronald B Williams, June Yamrozik, Robert W Biederman

**Affiliations:** 1 Cardiac MRI, Allegheny General Hospital, Pittsburgh, PA, USA

## Background

Cardiac magnetic resonance (CMR) imaging generally allows more accurate and valid quantification of cardiac function, mass and regurgitant volumes than echocardiography. Recent technological advancements in CMR have made evaluation of cardiac valves more reliable. However, there are no studies in the literature that compare semi-quantitative grading (SQG) using CMR SSFP sequences with quantitative grading (QG) based on stroke volumes and phase velocity mapping (PVM).We propose SQG of cardiac valvular regurgitations based on CMR SSFP sequences is feasible and highly correlative with standard CMR QG methods.

## Methods

CMR data between Jan 2007 to Dec 2011 was prospectively evaluated for valvular regurgitant lesions. Patients were included if they had right and left ventricular volumetrics based on CMR SSFP sequences and PVM across the aortic and pulmonic valves with reported regurgitant volumes and fractions. Patients were excluded if they had prosthetic valves, cardiac arrhythmias and intra-cardiac shunts. Regurgitant lesions were semi-quantitatively (visually) graded on a standard scale of 0 to 4 (trace, mild, moderate, moderate to severe and severe) and compared with quantitative regurgitant fractions. Correlations were evaluated by Spearman's rho formula and kappa (κ) for intra and inter observer variability were obtained on 30% of study sample.

## Results

Ninety-seven, (n=97) patients representing 134 valvular regurgitations (MR, AR, TR, and PR) were analyzed by semi-quantitative and quantitative methods. There were 58 males (60%) and the average age of the patients was 55±18 years. There were 44 mitral, 50 aortic, 29 tricuspid and 11 pulmonary regurgitant lesions. The correlation between SQR vs. QG yielded the following results: 0.67 p < 0.001(MR, r=0.66, p<0.001; AR, r=0.68, p<0.002; TR, 0.68, p=0.001; PR=0.70, p=0.017). The results for QG vs. SQG accounting for clinically significant differences of ± 1 grade for the group were as follows: 0.95 p<0.001 (MR, r=0.91, p<0.001; AR, r=0.96, p<0.001; TR, r=0.99, p<0.001; PR, r=0.93, p<0.001). No discrepancy between surgical regurgitation was present (3+ vs. 4+). Weighted kappa results were 0.67 and 0.61 for intra and inter observer variability representing good correlation.

## Conclusions

Visual assessment of cardiac regurgitant lesions is reliable, accurate and reproducible when compared to formal quantitative analysis via CMR confirming robust role for CMR in assessing regurgitant lesions particularly when assessing for surgical decision-making.

## Funding

None

